# Genetic Determinants of Resistance among ESBL-Producing *Enterobacteriaceae* in Community and Hospital Settings in East, Central, and Southern Africa: A Systematic Review and Meta-Analysis of Prevalence

**DOI:** 10.1155/2021/5153237

**Published:** 2021-06-02

**Authors:** Onduru G. Onduru, Rajhab S. Mkakosya, Said Aboud, Susan F. Rumisha

**Affiliations:** ^1^Department of Microbiology, College of Medicine, University of Malawi, Private Bag 360, Zomba, Malawi; ^2^Department of Microbiology and Immunology, Muhimbili University of Health and Allied Sciences, P.O. Box 65001, Dar es Salaam, Tanzania; ^3^Directorate of Information Technology and Communication, National Institute for Medical Research, P.O. Box 9653, Dar es Salaam, Tanzania

## Abstract

**Background:**

The world prevalence of community and hospital-acquired extended-spectrum *β-*lactamase (ESBL)-producing *Enterobacteriaceae* is increasing tremendously. Bacteria harboring ESBLs are currently the number one critical pathogens posing a major threat to human health.

**Objective:**

To provide a summary of molecular evidence on the prevalence of ESBL-producing *Enterobacteriaceae* (ESBL-E) and associated genes at community and hospital settings in East, Central, and Southern African countries.

**Methods:**

We conducted a systematic literature search on PubMed and Google Scholar databases for the available molecular studies on ESBL-E in hospitals and community settings in East, Central, and Sothern Africa (ECSA). Published studies in English language involving gene characterization of ESBLs from human samples in hospital and community settings were included in the review, inception to November 2019. A random effect meta-analysis was performed to estimate the prevalence of ESBL-E.

**Results:**

A total of 27 studies involving molecular characterization of resistance genes from 20,225 ESBL-E isolates were included in the analysis. Seventy-four percent of all studies were hospital based, 15% were based in community settings, and others were done in both hospital and community settings. Of all the studies, 63% reported *E. coli* as the dominant isolate among ESBL-E recovered from clinical samples and *Klebsiella pneumoniae* was reported dominant isolates in 33% of all studies. A random pooled prevalence of ESBL-E was 38% (95% CI = 24–53%), highest in Congo, 92% (95% CI = 90–94%), and lowest in Zimbabwe, 14% (95% CI = 9–20%). Prevalence was higher in hospital settings 41% (95% CI = 23–58%) compared to community settings 34% (95% CI = 8–60%). ESBL genes detected from clinical isolates with ESBL-E phenotypes in ECSA were those of Ambler molecular class A [1] that belongs to both functional groups 2be and 2d of Bush and Jacob classification of 2010 [2]. Majority of studies (*n* = 22, 81.5%) reported predominance of *bla*CTX-M gene among isolates, particularly CTX-M-15. Predictors of ESBL-E included increased age, hospital admissions, previous use of antibiotics, and paramedical use of herbs.

**Conclusion:**

Few studies have been conducted at a molecular level to understand the genetic basis of increased resistance among members of ESBL-E in ECSA. Limited molecular studies in the ECSA region leave a gap in estimating the burden and risk posed by the carriage of ESBL genes in these countries. We found a high prevalence of ESBL-E most carrying CTX-M enzyme in ECSA with a greater variation between countries. This could be an important call for combined (regional or global) efforts to combat the problem of antimicrobial resistance (AMR) in the region. Antibiotic use and hospital admission increased the carriage of ESBL-E, while poor people contributed little to the increase of AMR due to lack of access and failure to meet the cost of healthcare compared to high income individuals.

## 1. Introduction

Pathogenic bacteria evolve to resist the actions of antimicrobials through acquired and intrinsic mechanisms including production of *β*-lactamase enzymes, which inactivates antibiotic and decreases its therapeutic value [1, 2]. Extended-spectrum *β*-lactamases produced by many gram-negative bacteria, mostly *Enterobacteriaceae*, are able to hydrolyze penicillins, cephalosporin, and monobactams . They are mostly effective against a range of *β*-lactam drugs including ceftazidime, ceftriaxone, cefotaxime, and aztreonam [3–5]. In many cases, resistance to these antibiotics is transferred among bacteria through gene transfer systems of mobile genetic elements carried in bacteria plasmids or transposons by bacterial recombination process that involves conjugation, transformation, and transduction.

The world prevalence of community and hospital-acquired ESBL-E is increasing tremendously. Bacteria harboring ESBL enzymes are currently the number one critical pathogens posing a major threat to human health [6]. The spread and dissemination of infections caused by ESBL-E are associated with increased morbidity and mortality, health care costs, the need for development of new wide-spectrum antimicrobials and lengthy hospital stay of infected patients. This is because of a major decrease in therapeutic value of mostly used drugs as a result of resistance [7–11].

There has been a significant advancement in the understanding of ESBL-producing bacteria epidemic which was previously related to hospital-acquired infections [12, 13]. Recent increased recovery of ESBL-E from community and environmental samples [14–16], especially *E. coli* commonly causing community acquired urinary tract infections (UTIs) [17], indicates a probability of the shift of importation of ESBL-producing bacteria to hospitals rather than vice versa. The spread of community acquired ESBL carrying pathogens is accelerated by between-persons transmission of ESBL bacteria in the communities. Some studies suggest there are significantly higher transmission rates of ESBL-producing bacteria among community households as compared to hospital transmissions [18]. However, detailed studies describing the ESBL-E reservoirs and transmission routes in diverse settings are still limited.

In most poor resource countries of East, Central, and Southern Africa, there is lack of routine surveillance systems that could estimate the magnitude and risk factors as well as clinical outcomes associated with ESBL *Enterobacteriaceae* [19]. The overuse and misuse of antimicrobial agents in the environmental sector, agriculture, and human and veterinary medicine propel the spread of antimicrobial resistance among infectious bacteria. Other factors such as easy access to antibiotics, weak health systems, environmental contaminations, poor hygiene and sanitation services or practices, incomplete decontamination of medical devices, and lack of laboratory capacities for pathogens detection and surveillance have been described as important drives of the increasing resistance among members of ESBL *Enterobacteriaceae* in the region [20–22].

### 1.1. Objective

To better understand genetic determinants of resistance among ESBL-producing *Enterobacteriaceae* in East, Central, and Sothern Africa, we sought to summarize molecular evidence on the prevalence of ESBL-E and associated genes at community and hospital settings.

## 2. Material and Methods

This review and meta-analysis of prevalence has been conducted in compliance with Preferred Reporting Items for Systematic Reviews and Meta-Analyses (PRISMA) statement and checklist [23].

### 2.1. The Study Area

The review provides information on published articles from East, Central, and Southern Africa (ECSA) countries in accordance with Africa Union Countries profile [24]. We included six countries from the eastern Africa which are Tanzania, Kenya, Uganda, Rwanda, Burundi, and Ethiopia; five countries from central Africa, Republic of Chad, Central African Republic, Democratic Republic of Congo, Republic of the Congo, and Gabon; and Seven southern African countries of Botswana, Angola, South Africa, Malawi, Namibia, Zambia, and Zimbabwe.

### 2.2. Literature Search

Literature search of peer reviewed studies was conducted on PubMed and Google Scholar databases for the available molecular studies on ESBL-E in hospitals and community settings in ECSA. The search strategy included the following index terms and Boolean operators **(**extended-spectrum beta-lactamase OR extended-spectrum OR beta-lactamase OR ESBL beta-lactamases OR beta-lactamase OR Enterobacteriaceae) AND Community OR Hospital AND (Botswana OR Burundi OR Central African Republic OR Chad OR Congo OR Democratic Republic Congo OR “Democratic Republic of the Congo” OR Zaire OR Ethiopia OR Gabon OR Kenya OR Malawi OR Rwanda OR South Africa OR “South Africa” OR Tanzania OR Uganda OR Zambia OR Zimbabwe OR Southern Africa OR Eastern Africa OR East Africa OR Central Africa).

### 2.3. Study Inclusion and Exclusion Criteria

All available published molecular studies (involving genotypic characterization of ESBL) in English language on human subject reporting ESBL-E in hospitals and communities in ECSA were considered eligible. Unpublished studies, editorials, letters, studies on nonhuman subjects, studies published in other languages than English, and studies that did not utilize molecular tools were excluded. All studies available on the selected databases at the time of data extraction were examined (inception to November 2019).

### 2.4. Data Extraction

Data extraction checklist was developed to guide the acquisition of the information which included name of the author(s), year of publication, study setting (community or hospital), study design, subjects/target population, source of isolates/specimen, clinical samples, sample size, bacteriological methods for estimating ESBL's, molecular methods used, isolate species, number of isolates analysed, ESBL positive isolates, genes encoding for ESBL identified and risk factors associated with ESBL infection if studied.

### 2.5. Data Analysis

Acquired data were entered into Excel spreadsheet and statistical analysis was done using Stata version 12 (STATA Corporation, College Station, TX, USA). We performed a random effect meta-analysis to determine heterogeneity of ESBL-E prevalence in ECSA. Decision to perform random effect meta-analysis over fixed effect meta-analysis was made due to an assumption that the difference in ESBL-E prevalence among studies in ECSA is attributed to different factors such as study settings (hospital and community) and different laboratory methods used for detection of ESBL-E. A new program in STATA (*Metanprop)* specific for pooling binomial data including methods of computation of the 95% confidence intervals (CI), continuity correct, and the Freeman–Tukey transformation was used [25]. The risk of publication bias was assessed using funnel plot and Begg's rank correlation test for funnel plot asymmetry [26].

## 3. Results

**Figure 1:**
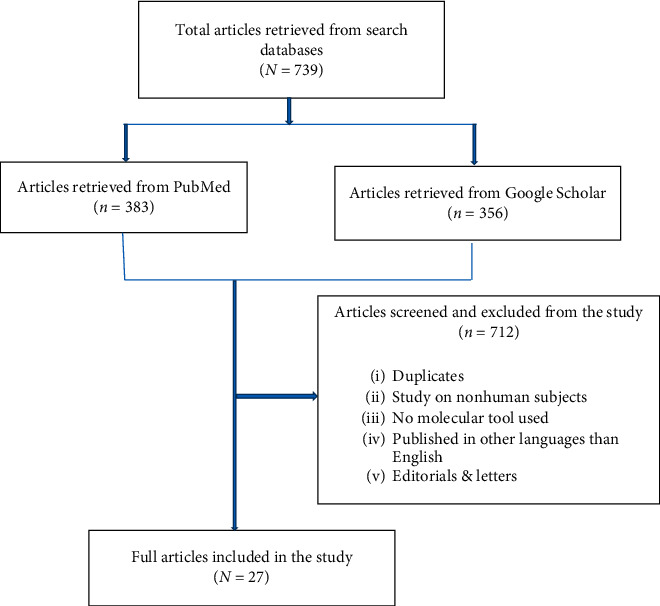
The flow of study selection process.

A total of 27 studies involving molecular characterization of ESBL-E that were retrieved from 11 countries in Eastern, Central, and Southern Africa region met the inclusion criteria and were included in this review (Figure 1). The publication year of the studies ranged between 2005 and 2019. Cross-sectional studies comprised 93% of all studies [9, 27–41]. Majority of studies (74%) were conducted in hospital settings, 15% in the community settings, and others in both hospital and community settings.

### 3.1. Bacteriological Methods for Estimating ESBL Producers

The most utilized method of ESBL estimation and confirmation was double disk synergy test (DDST) (59%); one study used both DDST and combination disk method (CD) [42]. An automated VITEX 2 systems was used in 22% of the studies [32, 35, 39, 40, 43, 44] while 15% used E-test [9, 41, 45, 46].

### 3.2. Molecular Tools Used for the Detection of ESBL Genes

Three molecular tools were used in all 27 studies to detect the presence of ESBL encoding genes among isolates. These included microarray, polymerase chain reaction (PCR), and sequencing. Seventeen studies (63%) used both PCR and sequencing, other studies either utilized PCR (22%) [9, 30, 33, 41, 46–48] or sequencing (11%) [22, 29, 49], and one study used microarray alone [32].

### 3.3. Country Prevalence of ESBL-Producing Enterobacteriaceae from Human in Eastern, Central, and Southern Africa

**Figure 2:**
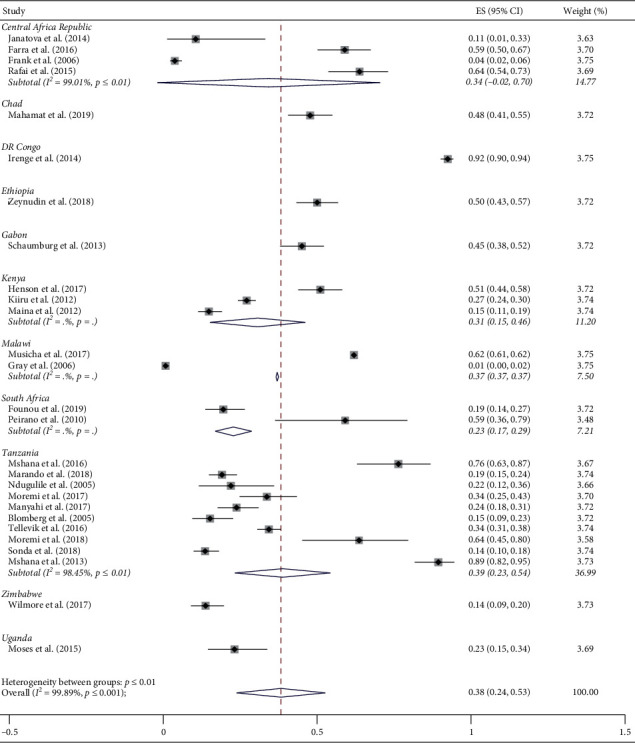
Individual study prevalence and pooled prevalence of ESBL-producing *Enterobacteriaceae* in East, Central, and Southern Africa (the red line indicates statistical significance of the results).

A total of 20,225 ESBL-producing isolates from various clinical samples were reported in 27 studies across Eastern, Central, and Southern Africa. Eighty-one percent of the studies isolated both *Klebsiella pneumoniae (K. pneumoniae)* and *Escherichia coli* (*E. coli*). Of the studies, 63% reported predominance of *E. coli* among isolates, *Klebsiella pneumoniae* was dominant in 33%, and 5% of the studies reported that *Proteus* spp. was dominant. Other *Enterobacteriaceae* isolated in a small proportion that could not dominate were *Enterobacter* spp., *Citrobacter* spp., *Morganella* spp., and *Providencia* spp. Prevalence of ESBL-E varied across the countries in ECSA; the highest prevalence was observed in DR Congo followed by Tanzania and Malawi at the rate of 92% (95% CI = 90–94%) [29], 89% (95%, CI = 82–95%), [50] and 62%(95%, CI = 61–62%) [51], respectively. The lowest individual study prevalence was reported in Zimbabwe [34] (Figure 2).

### 3.4. Pooled Prevalence of ESBL-Producing *Enterobacteriaceae* in East, Central, and Southern Africa

A random pooled prevalence of ESBL-producing *Enterobacteriaceae* in human from 11 ECSA countries with available data was 38% (95% CI = 24–53%). The pooled prevalence of ESBL-E in hospital settings was 41% (95% CI = 23–58%) and 34% (95% CI = 8–60%) in community settings. The variation in the prevalence of ESBL-E attributable to heterogeneity in ECSA was very high (I^2^ = 99.89%; *p* ≤ 0.001) (Figure 2).

### 3.5. Epidemiology of Extended-Spectrum *β*-Lactamases in ECSA

Genes encoding ESBL enzymes detected from clinical isolates with ESBL-E phenotypes in ECSA were those of Ambler molecular class A [1] that belongs to both functional groups 2be and 2d of Bush and Jacob classification (2010) [2]. These included SHV, CTX-M & TEM (*β*-lactamases of subgroups 2b, 2be, and 2ber), and OXA like *β*-lactamases of subgroup 2d (Table 1).

Majority of studies (*n* = 22, 82%) reported predominance of *bla*CTX-M genes among isolates particularly CTX-M-15, predominance of SHV gene was reported in three studies (*n* = 3, 11%) [9, 37, 45], and 7% reported equal proportion of TEM and CTX-M genes detected [49]. The detection of CTX-M genes was mostly on isolates from stool, rectal swabs, and wound swabs; other extended-spectrum *β*-lactamase coding genes including SHV, TEM, and OXA type were mostly detected in *K. pneumoniae* commonly recovered from blood samples [45, 46, 48, 49].

### 3.6. Factors Associated with ESBL-E Infections in ECSA

Five of 27 articles reviewed stressed out factors associated with ESBL-E infections to include increased age, hospital admissions, and previous use of antibiotic. A study in Gabon found that hospitalization and at least 5 years of age were the risks for the carriage of ESBL *Enterobacteriaceae* in children [43]. Higher ESBL-E carriage was strongly associated with treatment of HIV infection in Zimbabwean children [34]. In an area where antibiotics could not be afforded due to poverty and civil war, ESBL-E carriage in children was associated with high income families [52]. Increased number of children in the household, high median age, previous use of antibiotics, and use of local herbs for paramedical purposes were also predictors of ESBL-E carriage [36–40].

## 4. Discussion

In the present review, we present the summary of molecular determinants and the pooled prevalence of ESBL-producing *Enterobacteriaceae* in Eastern, Central, and Southern Africa. Few studies have been conducted to determine ESBL genes in these countries. Tanzania did most of the genotypic characterization of ESBLs while in other ECSA countries there was no any molecular work on AMR due to ESBL production by members of *Enterobacteriaceae*. The overall pooled prevalence was high (38%), close to previous findings by Sonda et al., [54] but higher than that found by Tansarli et al. [19] and Bulabula et al. [55].

According to the data generated from the present review, there was low pooled prevalence of ESBL-E in Zimbabwe and higher in DR Congo, Tanzania, and Malawi. Comparing our study with previous findings, it is surprising to find the prevalence of ESBL-producing pathogens in Malawi has increased by 89% within 12 years since the introduction of third-generation cephalosporins. The rate of resistance due to ESBL production at the time of cephalosporins introduction in Malawi in 2005 was very low (0.07%); the reason for low prevalence at that time was tight restriction on the use of antibiotics [45]. However, the rate rose rapidly to 92% in 2017 indicating either misuse of third-cephalosporin drugs or loosened restrictions on the antibiotic use [51].

The prevalence of ESBL-E was higher in the hospital settings than the community settings; this was similar to finding of others [19, 56]. We noted that majority of isolates were obtained from cultures of patients with nosocomial infections leading to the higher rate of ESBL genes detection in hospital settings. Similarly, it has been described elsewhere that most nosocomial infections are associated with ESBL-producing bacteria [9, 22, 37, 43, 49, 57]. Our findings complement previous review by Tansarli et al. [19]. These findings can suggest possible influx of ESBL pathogens into the communities from the hospitals. However, bringing together various disciplines to collectively investigate the burden of ESBL pathogens in the environment, human, and animals interface could provide information on the contribution of these different reservoirs to the ESBL-E epidemic.

Higher prevalence of ESBL-E in ECSA according to this review could be attributed to some factors including the focus of the studies reviewed and resistant genes targeted. Many studies across these countries reporting ESBL-E from human either were focused on certain ESBL bacteria, mainly *E. coli* and *K. pneumoniae* [22, 42, 49], or targeted the detection of specific ESBL genes [32, 35, 38, 40, 41]. Therefore, there were high chances of escalating the prevalence of ESBL producers without inclusion of other *β*-lactamases in the studies. However, there could also be inadequate infection control in healthcare systems contributing to the increased spread of ESBL-E in these poor resource countries as previously suggested [58]. Selective pressure due to heavy use of *β*-lactam antibiotics as a first-line treatment of infections caused by *Enterobacteriaceae* has been described to increase the spread of resistance and hence could lead to high prevalence in ECSA [32, 42]. Increased carriage of ESBL-E has also been associated with the use of local herbs such as *Aloe vera* and other herbal extracts used for paramedical purposes [35]. In most instances, poverty has been linked with increased incidence of ESBL-E. However, our study suggests that people who are poor contribute little to the increased spread of resistance among member of ESBL-E compared to those who can afford to buy and use antibiotics. Since majority of the studies were from hospital settings, it could be that the poor are not well represented due to either lack of access or failure to meet the cost of healthcare.

The fact that few molecular studies have been conducted in the ECSA region undermines the understanding of ESBL gene diversity implicated in hospital and community acquired infections caused by ESBL-E strains. Most studies done with regard to ESBL-E in the region looked at identifying the pathogens involved and phenotypic resistance patterns rather than the genes responsible for such resistance. Similar to other studies [59, 60], we found CTX-M genes particularly CTX-M-15 dominating other types of ESBL genes in both hospital and community isolates. With few allelic groups of ESBLs reported in the reviewed studies, our findings indicated that CTX-M-15 is more common than other ESBL types found in both clinical and nonclinical samples [35, 38, 40, 41, 60–63]. Previous studies have suggested that commensal isolates originating from infection of the gut and intestines carried different ESBL genes depending on bacteria species, plasmids, and location of the chromosome bearing the resistant gene [33]. However, in this study we observed invariability of ESBL gene types between community and hospital isolates across the studies.

The importance of genotypic detection of ESBL genes in bacteria over phenotypic screening cannot be ignored; this is because these genes are carried on plasmids permitting higher chances of horizontal transfer which cannot be detected phenotypically. Phenotypic screening for ESBL pathogens is useful for surveillance purposes but they can underestimate the burden of resistance posed by ESBL pathogens. Previous reports detected more resistant ESBL producers when molecular methods were used compared to phenotypic methods [27]. For definitive therapy selection, relying on phenotypic determination of ESBL enzymes alone may not be sufficient. Therefore, molecular detection is inevitable.

### 4.1. Strengths and Limitations

One of the strengths of this review is that we included studies published in English which is the official language of many countries in the study area. Searching Google Scholar database provided an advantage to retrieve articles that could have not been published in highly indexed journals. Institutional subscription also allowed retrieval of articles that were not available for free access. However, the study was limited by the challenge encountered in determining true prevalence because some studies were laboratory surveillance based and did not provide the actual sample size or number of participants from which the clinical samples were drawn.

### 4.2. Conclusion and Recommendations

Few studies have been conducted at a molecular level to understand the genetic basis of increased resistance among members of ESBL-E in ECSA. Limited molecular studies in the ECSA region leave a gap in estimating the burden and risk posed by the carriage of ESBL genes in these countries. We found a high prevalence of ESBL-E most carrying CTX-M enzyme in ECSA with a greater variation between countries. This could be an important call for combined (regional or global) efforts to combat the problem of AMR in the region. Antibiotic use and hospital admission increased the carriage of ESBL-E, while poor people contributed little to the increase of AMR due to lack of access and failure to meet the cost of healthcare compared to high income individuals.

## Figures and Tables

**Table 1:** Prevalence of ESBL-producing *Enterobacteriaceae* from human in Eastern, Central, and Southern Africa.

Number of article (s)	Country	Settings	Prevalence (%)	ESBL gene(s)	Reference
3	Central Africa Republic	Hospital	59	*bla*CTX-M-15	[52]
		Community	11	*bla*CTX-M-15, *bla*TEM-1, *bla*OXA-1, *bla*SHV-2a, & *bla*SHV-62	[33]
			4	*bla*CTX-M	[31]
1	Chad	Hospital	48	*bla*CTX-M-9, *bla*CTX-M-14, *bla*CTX-M-15, *bla*CTX-M-27, *bla*TEM-1, & *bla*OXA-1	[30]
1	DR Congo	Hospital	92	*bla*CTX-M-1	[29]
1	Ethiopia	Hospital	50	*bla*CTX-M-15, *bla*SHV, & *bla*TEM	[32]
1	Gabon	Hospital	45	*bla*CTX-M-15, *bla*CTX-M-8, *bla*CTX-M-1, & *bla*TEM	[43]
3	Kenya	Hospital	51	*bla*CTX-M-15, *bla*SHV-2, *bla*SHV-12 , *bla*SHV-27, *bla*OXA-1, & *bla*OXA-10	[22]
		Community	15	*bla*SHV, *bla*CTX-M, & *bla*TEM	[46]
		Hospital & community	27	*bla*SHV-5, *bla*SHV-12, *bla*SHV-52, *bla*CTX-M-8, *bla*CTX-M-14, *bla*CTX-M-9, *bla*CTX-M-15, *bla*CTX-M-3, *bla*CTX-M-1, *bla*TEM-12, *bla*TEM-125, *bla*TEM-50, *bla*TEM-78, *bla*TEM-109, *bla*TEM-152, & *bla*TEM-158	[42]
2	Malawi	Hospital	62	*bla*CTX-M	[51]
			1	*bla*SHV-11, *bla*SHV-12, *bla*SHV-27, *bla*CTX-M-15, & *bla*TEM-63	[45]
2	South Africa	Hospital	19	*bla*CTX-M-15, *bla*TEM-1b, *bla*SHV-1, & *bla*OXA-1	[49]
			59	*bla*CTX-M-3, *bla*CTX-M-14, *bla*CTX-M-15, & *bla*SHV-2	[44]
10	Tanzania	Community	76	*bla*CTX-M-15	[40]
			34	*bla*CTX-M-9, *bla*CTX-M-15, *bla*CTX-M-55, blaTEM-1, *bla*SHV-1, & *bla*SHV-11	[35]
		Hospital & community	24	*bla*CTX-M-15 & *bla*SHV-12	[41]
			34	*bla*CTX-M-14, *bla*CTX-M-15, *bla*SHV-5, & *bla*SHV-12	[53]
		Hospital	64	*bla*CTX-M-15	[39]
			14	*bla*CTX-M	[38]
			89	*bla*CTX-M-15, *bla*SHV-11, *bla*TEM-1, *bla*TEM-104, & *bla*TEM-176	[50]
			19	*bla*SHV-1, *bla*SHV-11, *bla*SHV-27, *bla*SHV-33, *bla*CTX-M-15, *bla*TEM-1B, & *bla*OXA-1	[36]
			22	*bla*SHV-12, *bla*SHV-28, *bla*CTX-M-15, & *bla*TEM-1	[37]
			15	*bla*SHV-2a, *bla*SHV-12, *bla*CTX-M-15, & *bla*TEM-63	[9]
1	Uganda	Hospital	23.2	*bla*SHV, *bla*CTX-M, & *bla*TEM	[27]
1	Zimbabwe	Hospital	14	*bla*CTX-M-3, *bla*CTX-M-14, *bla*CTX-M-15, *bla*CTX-M-27, *bla*TEM, & *bla*SHV	[34]
